# CNX-013-B2, a unique pan tissue acting rexinoid, modulates several nuclear receptors and controls multiple risk factors of the metabolic syndrome without risk of hypertriglyceridemia, hepatomegaly and body weight gain in animal models

**DOI:** 10.1186/1758-5996-6-83

**Published:** 2014-08-12

**Authors:** Manoj Kumar Sadasivuni, Bobbili Madhusudhan Reddy, Jaideep Singh, Mammen O Anup, Venkategowda Sunil, Mudigere N Lakshmi, Sivakumaran Yogeshwari, Suni K Chacko, Talanki Lokesh Pooja, Anilkumar Dandu, Chandrashekaran Harish, Aralakuppe S Gopala, Shivakumar Pratibha, Baisani S Naveenkumar, Puttrevana M Pallavi, Mahesh Kumar Verma, Yoganand Moolemath, Baggavalli P Somesh, Marikunte V Venkataranganna, Madanahalli R Jagannath

**Affiliations:** Connexios Life Sciences Pvt Ltd, Bangalore, India

**Keywords:** Diabetes, Dyslipidemia, PPAR pan-activator, Insulin sensitizer, Exercise mimetic, CHF, Steatosis

## Abstract

**Background:**

In addition to their role in growth, cellular differentiation and homeostasis Retinoid X Receptors (RXR) regulate multiple physiological and metabolic pathways in various organs that have beneficial glucose and lipid (cholesterol) lowering, insulin sensitizing and anti-obesity effects. Rexinoids, compounds that specifically binds and activate RXR, are therefore considered as potential therapeutics for treating metabolic syndrome. Apparently many of the rexinoids developed in the past increased triglycerides, caused hepatomegaly and also suppressed the thyroid hormone axis. The aim of this study is to evaluate CNX-013-B2, a potent and highly selective rexinoid, for its potential to treat multiple risk factors of the metabolic syndrome.

**Methods:**

CNX-013-B2 was selected in a screening system designed to identify compounds that selectively activated only a chosen sub-set of heterodimer partners of RXR of importance to treat insulin resistance. Male C57BL/6j mice (n = 10) on high fat diet (HFD) and 16 week old ob/ob mice (n = 8) were treated orally with CNX-013-B2 (10 mg/kg twice daily) or vehicle for 10 weeks and 4 weeks respectively. Measurement of plasma glucose, triglyceride, cholesterol including LDL-C, glycerol, free fatty acids, feed intake, body weight, oral glucose tolerance and non-shivering thermogenesis were performed at selected time points. After study termination such measurements as organ weight, triglyceride content, mRNA levels, protein phosphorylation along with histological analysis were performed.

**Results:**

CNX-013-B2 selectively activates PPARs- α, β/δ and γ and modulates activity of LXR, THR and FXR. In ob/ob mice a significant reduction of 25% in fed glucose (p < 0.001 ), a 14% (p < 0.05) reduction in serum total cholesterol and 18% decrease (p < 0.01) in LDL-C and in DIO mice a reduction of 12% (p < 0.01 ) in fasting glucose, 20% in fed triglyceride (p < 0.01) and total cholesterol (p < 0.001) levels, coupled with enhanced insulin sensitivity, cold induced thermogenesis and 7% reduction in body weight were observed.

**Conclusion:**

CNX-013-B2 is an orally bio available selective rexinoid that can be used as a novel therapeutic agent for management of multiple risk factors of the metabolic syndrome without the risk of side effects reported to be associated with rexinoids.

**Electronic supplementary material:**

The online version of this article (doi:10.1186/1758-5996-6-83) contains supplementary material, which is available to authorized users.

## Introduction

Metabolic syndrome is a cluster of risk factors comprising raised plasma glucose, abdominal obesity and high blood pressure all of which increase mortality due to cardiovascular disease. Management of metabolic syndrome includes behavioural changes aimed at promoting weight loss through dietary modifications and exercise and a combination of therapies directed to reduce specific metabolic risk factors [1]. In addition to diabetic dyslipidemia and insulin resistance such others factors as inflammation, oxidative stress, enhanced matrix metalloproteinase activity, activation of local renin angiotensin system and the accumulation of advanced glycation end-products contribute to the development and progression of macrovascular disease in diabetes.

Peroxisome Proliferator-Activated Receptors (PPAR), members of the nuclear receptor family of transcription factors, participates in molecular pathways that can modify a host of aforesaid biochemical pathways [2–6]. PPARγ agonists, rosiglitazone and pioglitazone enhance insulin sensitivity, lower hyperglycemia and free fatty acid concentrations by improving glucose and lipid metabolism, improves adipokine profile and reduce adipose tissue inflammation [7–9]. PPARα agonists, Fibrates, lower triglycerides, increase HDL-cholesterol, normalise low density lipoprotein size distribution and also display anti-inflammatory and anti-atherosclerotic effects [9–12]. Activation of PPARδ in preclinical studies has displayed potential to control weight gain, enhance physical endurance, improve insulin sensitivity and ameliorate atherosclerosis [13]. The adverse side effects of rosiglitazone include weight gain, bone fractures, fluid retention, edema congestive heart failure all leading to myocardial infarction and ischemic cardiovascular events. On the other hand pioglitazone induces weight gain, increases fluid retention and possibly increased fractures and bladder cancer. The adverse side effects associated with fibrates include elevated serum creatinine, myopathy and rhabdomyolysis [9].

Attempts to improve safety profile of PPAR agonists have looked at balancing the relative potency and/or activity towards PPARα or PPARγ and also the selectivity/potency of cofactor recruitment as both these traits would likely demonstrate high levels of efficacy but with improved safety profile. Dual PPAR agonists were developed to combine the beneficial effects of PPARα and PPARγ for addressing CV risk in patients with T2DM. Muraglitazar, a dual PPARα/γ agonist with greater potency towards PPARγ, improved HbA1c and lipid profile but caused higher edema, CHF and CV deaths and development was subsequently stopped. Tesaglitazar, a dual PPARα/γ agonist with greated potency towards PPARα as against PPARγ was discontinued due to increase in body weight, edema and serum creatinine levels [9]. Recently development of Aleglitazar, a balanced activator of PPARα and PPARγ was discontinued in phase III due to safety signal and lack of efficacy.

It became clear that in the activation of PPARγ by a full agonist the dose response curve relation for multiple activities appeared to be linked while in case of a selective modulator the dose response relations between different activities were completely uncoupled. Selective peroxisome proliferator-activated receptor γ modulators (SPPARM) that display potent and highly efficacious insulin sensitization but low potency for side effects are being developed [14]. While many SPPSRMs are in preclinical development INT131 has displayed lowering of plasma glucose without typical thioazolidindione side effects in patients with T2DM [15].

Simultaneous activation of PPARα, PPARδ and PPARγ by a single compound is being pursued to treat the multiple defects associated with insulin resistance, type 2 diabetes and the metabolic syndrome [16]. Currently bezafibrate is reported to operate as a pan-agonist of all the three PPAR isoforms and has been effective in reducing insulin resistance, glucose, HbA1c, small dense LDL particles, atherosclerotic plaque regression and improves endothelial function [17].

LXR agonists display significant anti-diabetic activities in diabetic rodent models but are associated with the risk of hypertriglyceridemia and liver steatosis [18] while synthetic selective thyroid hormone (TH) receptor (TR) modulators (STRM) reduce dyslipidemia, obesity, fatty liver, and insulin resistance in preclinical animal models [19].

Retinoid X Receptors (RXR) are members of the nuclear receptor family of transcription factors that function as a ‘sensor’ receptor by binding specific liphophilic ligand and modulating gene expression. RXRs are considered ‘promiscuous’ as they form heterodimers with several other nuclear receptor family members. In addition RXRs are also known to exist as homodimers and homotetramers which can control their own signaling pathways. Binding of RXR ligands to heterodimers are reported to enhance transcriptional activation by RXR partner receptors. Three genes encode the Retinoid X Receptors - RXRα, RXRβ and RXRγ. Expression of RXRα is predominant in liver, RXRβ in CNS and RXRγ in skeletal muscle and some regions of CNS [20].

RXRα appears to have an important role in development as germline mutations are *in utero* lethal while mice expressing single RXR allele (RXRβ^-/-^ or RXRγ^-/-^) are completely viable indicating functional redundancy [21]. Similarly hepatocytes specific inactivation RXRα produces strong phenotype indicating a major role for RXRα [22]. It is apparent that retinoic acid receptors display distinct functions inspite of common hetero-dimerization partners [20]. RXRα plays an important role in pathways modulating cholesterol, fatty acid, bile acid, steroid and xenobiotic metabolism and homeostasis [23] in liver and modulates adipogenesis and lipolysis [24] in adipocytes. RXRγ has been demonstrated to control gene expression to enhance insulin sensitization and glucose disposal, increase uptake and oxidation of saturated fatty acids, increase desaturation of fatty acids and regulate oxidative slow-twitch phenotype [25]. RXR agonists have also been shown to activate PPARα-inducible genes and lower triglycerides and raise HDL levels *in vivo* [26], reduce atherosclerosis in apoE knockout mice [27] and activate RXR:PPARγ heterodimer to reduce hyperglycemia, hypertriglyceridemia and hyperinsulinemia [28].

While rexinoids have demonstrated insulin sensitizing, glucose lowering and anti-obesity effects in animal models of disease they have been associated with undesirable effects as hypertriglyceridemia and suppression of the thyroid hormone axis both in animals and in humans [29]. It is the considered opinion of several investigators that a rexinoid that is selective and activates receptor complexes of benefit to insulin resistance could be of major therapeutic significance.

In this study we report the development of a heterodimer selective rexinoid, CNX-013-B2, that provides significant control of insulin resistance, hyperinsulinemia, glucose, lipid and body weight in mice models of disease. Specifically pharmacological effect of CNX-013-B2 is devoid of the commonly observed side effects associated with many PPARγ and RXR agonists. CNX-013-B2 is a safe and efficacious therapeutic with potential to compliment the current standard of care to provide robust and long term control of the various risk factors associated with the metabolic syndrome.

## Material and methods

### Reagents and kits

Includes Accu-check glucometer (Roche Diagnostics, Germany), Ultra-sensitive insulin ELISA kit (Crystal Chem Inc, USA), TAG estimation kit (Diasys Diagnostics system, Germany), FFA estimation kit (Randox Laboratories, UK), Cholesterol estimation kit (Diasys Diagnostics system, Germany, cat# 113009910704), Glycerol estimation kits (Sigma, cat#F6428). Triton-X and Fluromount were procured from Sigma-Aldrich. Bexarotene (Sigma).

### Transactivation assay

HEK-293 cells (ATCC) were seeded one day prior to transfection. For assessing EC50 of activation of RXR isoforms 2 μg of hRXR α, β, or γ (OriGENE, USA) over-expressing vector, 1 μg of plasmid expressing firefly luciferase under RARE element (RARE-Luc) and 25 ng of renilla luciferase vector (QIAGEN, USA) were co-transfected using lipofectamine reagent. For assessing transactivation of heterodimer partners 2 μg each of hRXRα/hLXRα, hRXRα/RARα, hRXRα/PPARα, hRXRα/PPARγ and hRXRγ/PPARδ (OriGENE, USA) were co-transfected alongwith 1 μg of plasmid expressing firefly luciferase under respective LXRE, XRE or PPRE element and 25 ng of renilla luciferase vector. After 24 h of transfection, cells were treated with different concentrations of CNX-013-B2 for 24 h followed by estimation of luciferase activity using Dual Luciferase Reporter Assay System (Promega). Luciferase activity was normalized to that of renilla luciferase. For EC50 determination, activation was measured at different concentration of CNX-013-B2 (1, 5, 10, 50, 100, 500, 1000 and 5000 nM). EC50 was calculated using Graphpad prism software.

#### Study in C57BL/6j mice on high fat diet

Six week old male C57BL/6 J mice were housed 2 per polypropylene cage, maintained at 23 ± 1°C, 60 ± 10% humidity, exposed to 12 hour cycles of light and dark and provided ad libitum access to either chow, 10% kcal from fat or high fat diet, HFD D12492, 60% kcal from fat (both from Research Diets, USA) and water. All the study protocols, animal maintenance and experimental procedures were approved by the Institutional Animal Ethics Committee (IAEC) of Connexios Life Sciences, which is according to the CPCSEA (Committee for the Purpose of Control and Supervision of Experiments on Animals) guidelines, Govt. of India. Post high fat feeding for 11 weeks animals were randomized to specific treatment groups based on body weight, glucose AUC during OGTT, fasting and fed blood glucose and fasting TG levels.

Animals (n = 10) in the lean control group were fed normal chow diet while animals in the treatment group (n = 10) and DIO control group (n = 10) were fed high fat diet throughout the experimental period. Animals in the treatment group received 10 mg/kg CNX-013-B2, twice daily, po as suspension in 1% MC (methyl cellulose) as vehicle. Lean control and DIO controls animals were administered only vehicle. Body weight (weekly) and feed consumption, as average for 2 animals (daily), were recorded. Blood collected from tail vein after 24 h of the previous dose was subjected to glucose and triglyceride estimation. oGTT was performed in 6 h fasted mice with 2 g/kg, of oral glucose challenge. After 10 weeks of treatment blood collected from retro-orbital bleeding was used for estimation of glycerol, free fatty acid, total cholesterol and LDL-C. Blood was collected in the fed state for cholesterol estimation and for estimation of all other parameters blood was collected in the fasting state. Animals were euthanized post 6 h fasting under isoflurane anaesthesia, necropsied and liver excised immediately, weighed and taken for estimation of triglyceride. Different adipose depots were separated and weighed.

#### Study in ob/ob mice

Male ob/ob and lean C57BL/6 J mice were procured from Harlan laboratories, acclimatized and fed a standard laboratory diet. The lean control and the ob/ob mice were 16 weeks old at the start of the study and were randomized to either vehicle or drug (CNX-013-B2- 10 mg/kg, BID, po) treatment groups based on body weight, fed glucose and glucose AUC determined in an oral glucose tolerance test (oGTT).

The animals were treated for 4 weeks and during the study body weight, food intake, fed glucose and fasting triglycerides were monitored at regular intervals. In an oGTT performed at end of the study glucose levels were determined at different time intervals post glucose load. Serum collected, before animals were euthanized under isoflurane anaesthesia, was used for estimation of glycerol, free fatty acid, cholesterol and LDL-C.

### Oral glucose tolerance test (oGTT)

CNX-013-B2 or vehicle was administered to 6 h fasted animals 30 min prior to administration of glucose (2 g/kg b. wt) by oral gavage. Blood samples collected from the tail vein 30 min before treatment and at 0, 15, 30, 60, 90, 120 and 180 min after glucose load was used for estimating plasma glucose and insulin.

### Measurement of thermogenesis

For assessing cold induced thermogenesis in DIO mice, animals were housed in a cold environment with an ambient temperature of 4°C and body temperature was determined every 15 min for a total of 75 min using a rectal probe. The animals were then shifted to room temperature and rectal temperature was measured at 10 minutes interval for a further 20 min period.

### Estimation of total cholesterol, LDL-C, glycerol and FFA

Blood collected by retro orbital route under Isoflurane anesthesia was allowed to clot for 30 min at room temperature, centrifuged at 10000 rpm for 10 minutes at 4°C and the serum was collected for further analysis. Serum total Cholesterol was measured using fully automated clinical chemistry analyzer EM360, (Transasia Bio-medicals Ltd) with ERBA Kits. LDLc was estimated by colorimetry using Diasys kit as per the manufacturer’s protocol. Glycerol was estimated by Colorimetric method using Sigma Kit and FFA by Randox kit.

### Estimation of tissue TG and cholesterol

Tissue TG and Cholesterol were extracted according to Folch’s method. Briefly, 1 ml of 10% tissue homogenate was extracted with 5 ml of chloroform: methanol (2:1) mixture, the organic layer was separated and dried in a speed vac. The residue was re-suspended in isopropyl alcohol and TG and cholesterol levels were estimated by using TAG kit and cholesterol kit respectively from Diasys (Diasys Diagnostics system, Germany).

#### Histochemical and histological analysis of liver

To examine morphology formalin-fixed liver samples were paraffin-embedded, sectioned at 5 μm and stained with hematoxylin and eosin (H&E). All slides were examined under light microscopy at low (10X), (40X) and high (100X) magnification. For glycogen staining, sections were deparaffinized, hydrated and immersed in periodic acid solution for 5 minutes at room temperature. After rinsing in distilled water, the sections were covered with Schiff’s reagent for 15 minutes at room temperature and washed in running tap water for 5 minutes. The sections were counterstained with Mayer’s hematoxylin for 90 seconds, rinsed in running tap water, dehydrated, cleared and mounted under DPX mountant.

##### Adipocyte size measurement

Formalin-fixed adipose tissue from various depots was paraffin-embedded, sectioned at 5 μm and stained with hematoxylin and eosin (H&E) and 10 different microscopic fields photographed at 400× magnification. Morphometry of the captured images was performed using ProgRes Pro, v.2.8.8 image analysis suite. The adipocytes were traced along their perimeter and area was calculated. The mean area of adipocytes from all the groups were statistically analysed using Graphpad Prism, v.5.0.

##### UCP1 expression in adipocytes

Formalin fixed paraffin embedded adipose tissue sections, from both inguinal and intra scapular brown adipose depots, were deparffinized in xylene and subjected to antigen retrieval in citrate buffer, followed by washing in buffer Triton X-Phosphate Buffered Saline (PBS) and blocked using 1% bovine serum albumin in PBS. The blocked tissue sections were incubated with anti-UCP1 primary antibody (1:50) for 1 hour. Alexa-Fluor 555 tagged secondary antibody (goat-anti-rat IgG, 1:100) was used for detection. After 45 minutes of incubation with secondary antibody, the sections were washed and wet mounted for examination under fluorescence microscope (Zeiss AX100). From immunofluorescence stained sections 30 different microscopic fields per sections were randomly selected. The images of each microscopic field were captured using ProgRes Pro, v.2.8.8 image analysis suite at a magnification 400X. The adipocytes positive for UCP1 expression were manually counted in all 30 microscopic fields and expressed as mean ± SEM.

### Measurement of succinate dehydrogenase activity

Gastrocnemius muscle pieces (10–12 mg/animal) were obtained from the animals and washed with KRBH media and incubated in 100 mM potassium phosphate buffer containing 50 mM sucrose, 10 mM sodium azide, 500 mM sodium succinate and 8 mM INT (Iodonitrotetrazolium chloride; Sigma) for 2 h. Muscle tissue samples without sodium succinate were used as negative control. After 2 h at 37°C, INT was dissolved in DMSO by vertexing and estimated at 644 nm. The difference in absorbance with/without succinate was calculated, normalized to total weight of muscle and represented as % control SDH activity.

### RNA isolation, reverse transcription and quantitative real time polymerase chain reaction (qPCR)

Total RNA was isolated from 100 mg of different tissues using Tri-reagent (Sigma, USA) as per manufacturer’s instructions and 2 ug of RNA was converted into cDNA by reverse transcription (ABI, USA) using the standard PCR method. Gene expression was measured using SYBR Green PCR Master Mix (Eurogenetic, Belgium) and relative levels of expression were quantified using 18S rRNA/ Beta actin/RPL13 as control housekeeping gene. The primer sequences for the genes analyzed are given in the supplementary table.

### Western blot

After 10 weeks of treatment of DIO mice on HFD and 4 weeks of treatment of ob/ob mice, animals in all experimental groups were sacrificed and 10 mg each of tissue samples was collected from muscle (gastrocnemius), adipose (mesenteric and inguinal) and liver from each animal. Lysates (50 μg each from liver, muscle and adipose) were prepared by homogenization and subjected to SDS-PAGE, transferred onto nitrocellulose membranes, probed with primary antibody against pPPAR-γ (Ser 273, Cell Signaling), β-actin, p-AKT, p-JNK, total JNK, IKK- β and total AKT (Cell signaling, USA) and developed by enhanced chemiluminescence (West Pico, Thermo Scientific, USA). The relative levels of p-AKT compared to Total AKT were quantified using Image J Ver. 4.2, NIH, Bethesda. For p-AKT measurement in mesenteric adipose tissue of DIO mice study, adipose tissues were incubated with or without 30nM insulin for 10 min. After the incubation period, tissues were processed for p-AKT measurement as mentioned above.

### Statistical analysis

All the values are expressed as Mean ± SEM; one way analysis of variance was performed followed by Dunnets, test for establishing the significance value of the treatment groups when compared with DIO control or ob/ob control. p < 0.05 was considered as statistically significant.

## Results

### CNX-013-B2 is a potent and selective rexinoid

HEK293 cells (Human embryonic Kidney cells) were used as model system to measure the transcriptional activation of transiently overexpressing RXR isoforms alone or RXR isoform co-transfected with LXRα or RARα or PPARα or PPARδ or PPARγ overexpressing vectors by CNX-013-B2. Reporter assay indicated that treatment with CNX-013-B2 almost uniformly activated all three RXR isoforms in a dose dependent manner and the calculated EC50 determined was 48nM, 66nM and 68nM towards RXRα, RXRδ and RXRγ respectively. Treatment with CNX-013-B2 enhanced transcriptional activity of RXR/PPAR isoforms and the EC50 for activation of RXR/PPARα, RXR/PPARδ and RXR/PPARγ was determined as 159nM, 178nM and 196nM respectively (Table 1). It is important to note that CNX-013-B2 did not activate RXR/LXR and RXR/RARα heterodimers (Additional file 1A and B). Also CNX-013-B2 did not activate any of the PPAR isoforms, α, δ or γ, in absence of RXR (Additional file 1C, D and E) while the PPARα, PPARδ and PPARγ were activated by their respective agonists namely fenofibrate, GW501516 and rosiglitazone. This indicates that PPAR-pan activity (Table 1) is a result of activation of RXR and we therefore consider CNX-013-B2 as a selective rexinoid with PPAR-pan activity. Due to non-availability of specific reagents we were not able to demonstrate impact of CNX-013-B2 on activation of RXR/FXR and RXR/TR heterodimer complexes. We have not performed binding studies with any of the nuclear receptors. In the RXR transfection assay Bexarotene (LG1069), a rexinoid approved for treating cutaneous T-cell lymphoma [30], displayed an EC50 of 18nM for activation of RXRα (Table 1). As the activation of the RXR/PPAR heterodimers by Bexarotene, in the cotransfection assays, was similar to that of CNX-013-B2 at 1 μM concentration (data not shown) we did not assess the EC50 towards individual RXR/PPAR isoform heterodimer complexes. However in contrast to CNX-013-B2 Bexarotene activated the RXR/LXR heterodimer complex (Additional file 1).

**Table 1:** **Potency towards RXR homedimers and heterodimers**

Nuclear receptors	CNX-013-B2	Bexarotene
	EC _50_(nM)	
RXRα	48	18
RXRβ	66	ND
RXRγ	68	ND
RXRα: PPARα	159	ND
RXRα: PPARδ	178	ND
RXRα: PPARγ	196	ND

### CNX-013-B2 significantly improves insulin sensitivity and glucose tolerance

**Figure 1:**
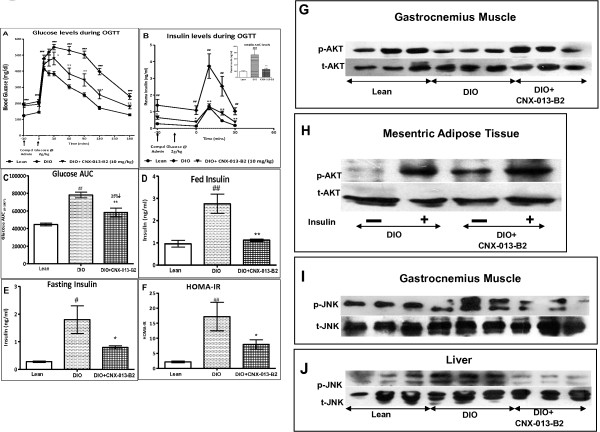
**CNX-013-B2 improves insulin sensitivity and glucose tolerance in C57BL6/j DIO mice on High Fat Diet. A**- OGTT in DIO on week 10 of treatment. Circle-Lean control, Diamond-DIO control and Triangle-CNX-013-B2 (10 mpk, bid). **B**- Insulin levels during OGTT. B insert- Insulin AUC, **C**- Glucose AUC 0–180 min, **D**- Fed insulin, **E**-fasting (6 h) and **F**-HOMA-IR. **G & H**- p-AKT levels in muscle and mesenteric adipose. **(I)**- p-JNK levels in gastrocnemius muscle. **(J)**- p-JNK levels in liver. All the values are expressed as Mean ± SEM; one way analysis of variance followed by Dunnett’s post hoc test for representing significance value of the treatment groups. P value significance was represented as (*) <0.05, (**) <0.01 and (***) <0.001 when compared with DIO mice. (#) <0.05, (##), <0.01 and (###) <0.001 as compared to lean mice.

**Figure 2:**
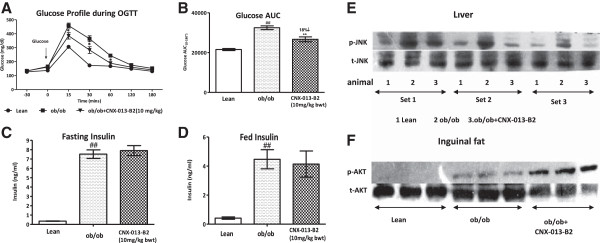
**CNX-013-B2 improves insulin sensitivity and glucose tolerance in ob/ob mice. A**- OGTT in ob/ob mice at week 4 of treatment. Circle-Lean, Square-ob/ob and Triangle-CNX-013-B2 (10 mpk, bid). **B**- glucose AUC (0–180 min), **C**- Fasting (6 h) insulin, **D**- fed insulin levels, **E**- p-JNK in liver, **F**- p-AKT levels in inguinal adipose. All the values are expressed as Mean ± SEM; one way analysis of variance followed by Dunnett’s post hoc test for representing significance value of the treatment groups. P value significance was represented as (*) <0.05, (**) <0.01 and (***) <0.001 when compared with ob/ob control. (#) <0.05 and (##) <0.01 as compared to ob/^+^.

To assess the impact of CNX-013-B2 on insulin sensitivity, oGTT, fasting and fed insulin levels and status of insulin signaling in muscle and adipose tissue were determined during the course of the study. In the OGTT performed in C57BL/6 J mice, the DIO control animals displayed significant glucose intolerance (Figure 1A), corresponding to a 75% increase in glucose AUC_0-180’_ (78134 ± 3235 vs 44596 ± 1492; p < 0.001) when compared to lean control animals on chow diet (Figure 1C). When compared with DIO control, animals treated with CNX-013-B2 for 10 weeks were significantly less insulin resistant and displayed enhanced glucose tolerance (78134 ± 3235 vs 58167 ± 4854; p < 0.01) corresponding to a 25% decrease in glucose AUC_0-180’_ (Figure 1C). Plasma Insulin levels in the first 30’ of oral glucose load was determined as another measure of insulin sensitivity. The DIO control animals were clearly insulin resistant and the 0–30’ insulin AUC (72.2 ± 17.05 vs 19 ± 1.93) was significantly higher (p < 0.001) compared to lean control animals (Figure 1B inset). The first phase insulin peak at 10’ time point in the DIO control animals was significantly higher (p < 0.01) than that in lean control animals (3.74 ± 0.73vs 1.29 ± 0.14 ng/ml; p < 0.01). In comparison with DIO control animals in CNX-013-B2 treated animals the insulin peak at 10’ time point was completely blunted (1.3 ± 0.18 vs 3.74 ± 0.73 ng/ml; p < 0.01), almost reaching levels observed in lean control animals (Figure 1B), resulting in a significant 73% reduction (Figure 1B inset) in the 0 – 30’ insulin AUC (72.2 ± 17.05 vs 19 ± 1.93). Even with reduced insulin secretion in the first 30’ CNX-013-B2 treated animals displayed a 29% reduction in the 30 – 180’ glucose AUC (45728 ± 4630vs 64361 ± 2943; p < 0.001) when compared with DIO control animals. Similarly CNX-013-B2 treated animals displayed a significant 60% decrease (Figure 1D) in fed insulin (1.12 ± 0.05 vs 2.76 ± 0.43 ng/ml; p < 0.01) levels and a 53% decrease (Figure 1E) in fasting insulin (0.8 ± 0.06 vs 1.8 ± 0.4 ng/ml; p < 0.05) as compared to DIO control at the end of the study. Accordingly the CNX-013-B2 treated animals showed a significant 55% reduction in HOMA-IR indicating significant improvement in whole body insulin sensitivity (Figure 1F). It is well known that AKT phosphorylation is reduced under conditions of insulin resistance and immediately restored upon onset of insulin sensitivity. Improvement in the insulin sensitivity was further supported by increased p-AKT levels in muscle (Figure 1G) and mesenteric adipose tissue (Figure 1H). Also treatment resulted in reduced p-JNK levels in skeletal muscle and liver indicating CNX-013-B2 reduces metabolic stress and hence improves insulin sensitivity (Figure 1I & J).Treatment of ob/ob animals with CNX-013-B2 at 10 mg/kg for 4 weeks improved insulin sensitivity and glucose tolerance resulting in an 18% decrease in glucose AUC (32464 ± 944 vs 26663 ± 1222; p < 0.01) (Figure 2A and B). However in these animals there was no decrease in either fasting or fed insulin levels (Figure 2C and D) respectively. In liver a significant reduction in p-JNK levels suggests robust reduction in overall hepatocyte metabolic stress (Figure 2E). A significant increase in p-AKT level was observed in adipocytes from inguinal depot in treated animals indicating enhanced insulin signaling (Figure 2F). It can be concluded that treatment with CNX-013-B2 enhances insulin sensitivity and improves glucose tolerance in ob/ob mice.

### CNX-013-B2 reduces serum glucose and lipid levels

**Figure 3:**
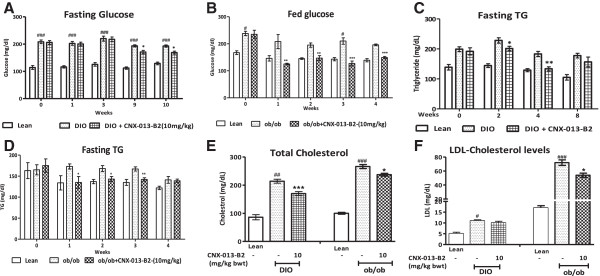
**CNX-013-B2 reduces blood glucose and lipid levels. A**- Fasting (6 h) glucose in DIO mice. **B**- Fed glucose in ob/ob mice. **C**- Fasting TG in DIO mice. **D**- Fasting TG in ob/ob mice. **E**- Total cholesterol and **F**-LDLc. All the values are expressed as Mean ± SEM; one way analysis of variance followed by Dunnett’s test for representing significance value of the treatment groups. P value significance was represented as (*) <0.05, (**) <0.01 and (***) <0.001 when compared with DIO control. (#) <0.05 and (###) <0.001 as compared to lean or ob/^+^.

At the start of the treatment period, fasting plasma glucose (FPG) levels were 208.38 mg/dl in the DIO control animals, 206.1 mg/dl in the CNX-013-B2 group and 114.13 mg/dl in the lean control animals on chow diet. After 3 weeks of dosing FPG levels started to decrease in the animals treated with CNX-013-B2 and reached a healthy and significant 14% decrease (197 ± 5 vs 169 ± 5 mg/dl; p < 0.01) by the end of the treatment period (Figure 3A). At the start of the study fed glucose levels had increased considerably and reached 238 ± 8 mg/dl in the animals designated as ob/ob control and 235 ± 15 mg/dl in the animals designated for treatment with CNX-013-B2 at 10 mg/kg. Compared to ob/ob control animals an acute and statistically significant 39% reduction in fed glucose levels (208 ± 26 vs 125 ± 3 mg/dl; p < 0.001) was recorded after one week of treatment itself and this trend continued and reached a statistically significant 24% reduction (196 ± 3 vs 149 ± 4 mg/dl; p < 0.001) at the end of 4 weeks (Figure 3B). After 8 weeks of high fat feeding, plasma triglyceride levels had reached 199 mg/dl in the DIO control group and 191.8 mg/dl in the group designated to be treated with CNX-013-B2 while in the lean control animals on chow diet it was 138.75 mg/dl. During the study circulating triglyceride levels remained consistently higher in the DIO control animals. Treatment with CNX-013-B2 caused a significant decrease in serum TG levels (Figure 3C) by the 4th week (183 ± 8 vs 133 ± 6 mg/dl; p < 0.01) while in the ob/ob animals the decrease in serum TG levels was not statistically significant (Figure 3D). In comparison with DIO control animals, treatment with CNX-013-B2 significantly reduced serum total cholesterol (214 ± 8 vs 170 ± 7 mg/dl; p < 0.001) in the fed state (Figure 3E) while the decrease in fasting LDL levels (11 ± 0.5 Vs 10 ± 0.6 mg/dl) was non-significant (Figure 3F). When compared with control ob/ob animals, in CNX-013-B2 treated animals both total cholesterol in the fed state (266 ± 7 vs 230 ± 5 mg/dl; p < 0.05;) and fasting LDL-C (72 ± 4 vs 54 ± 3 mg/dl; p < 0.05) decreased significantly (Figure 3E & F).

The reduction in serum glycerol and free fatty acids in both C57BL/6 J animals on DIO and in ob/ob mice was however statistically non-significant (Additional file 2A and B).

### CNX-013-B2 has significant effect on body weight

**Figure 4:**
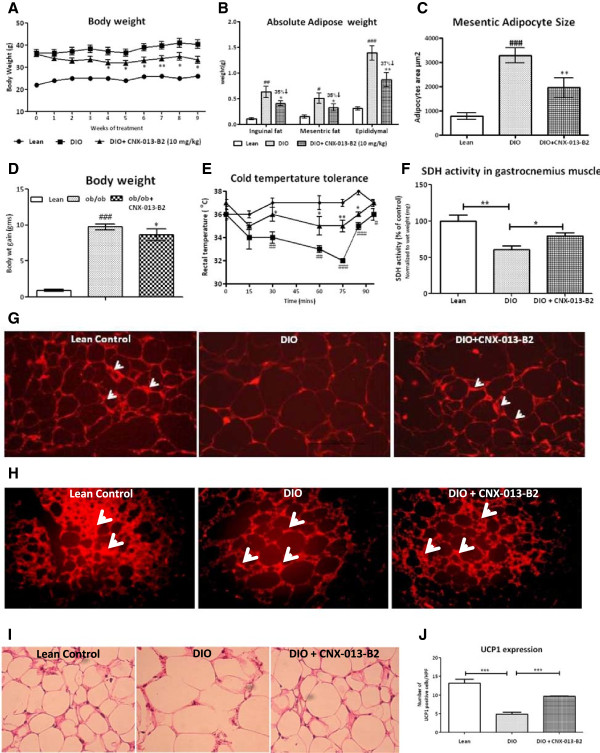
**CNX-013-B2 reduces body weight and enhances non-shivering thermogenesis. A**- Body weight in DIO mice, **B**-Absolute adipose depot weight, **C**- mesenteric adipocyte size, **D**- Body weight gain in ob/ob mice, **E**-.Cold temperature tolerance in DIO mice, **F**- SDH activity in gastrocnemius muscle of DIO mice, **G** and **H**-, UCPI expression levels in inguinal and brown adipocyte, **I**- H&E of adipocytes and **J**-UCP1 quantification in inguinal adipocytes. All the values are expressed as Mean ± SEM; one way analysis of variance followed by Dunnett’s test for representing significance value of the treatment groups. P value significance was represented as (*) <0.05, (**) <0.01 and (***) <0.001 when compared with DIO control. (#) <0.05, (##), <0.01 and (###) <0.001 as compared to lean or ob/^+^.

At the start of the study, the average body weight was 22 g, 36.41 g and 36 g in the lean control, DIO control group and in the CNX-013-B2 treatment group respectively. In the DIO control group, body weight increased from 36.41 g to 40.51 g during the 9 week study period. In contrast, beginning from the first week itself, treatment with CNX-013-B2 prevented any increase in body weight and instead a gradual decrease was observed all through the study and reached 33.43 g at the end of the treatment period. In effect a 7% decrease from basal body weight was recorded during the 9 weeks treatment (Figure 4A) and significant reductions in the absolute weight of inguinal (35%), mesenteric (35%) and epididymal (37%) fat depots were observed (Figure 4B). It is important to note that there was no change in food intake in the treated animals (data not shown). While the adipocytes from the mesenteric depot showed a significant (p < 0.01) decrease in size (Figure 4C) a non-significant decrease in size was observed in the adipocytes from the inguinal depot (data not shown). The decrease in body weight of ob/ob animals was non-significant after 4 weeks of treatment (Figure 4D) and a longer treatment period is perhaps necessary to demonstrate the effect on body weight in this genetic background.

### CNX-013-B2 enhances non-shivering thermogenesis

Basal body temperature was similar between lean control and DIO control animals. However, CNX-013-B2 treated animals recorded about 1°C higher temperature than DIO control animals (37 ± 0.3°C vs 36 ± 0.4°C). But 15’ after shifting to a 4°C environment, rectal temperature dropped by 2°C in both the DIO control and the CNX-013-B2 treated animals. Subsequently in contrast to the steady decrease that was observed in the DIO control animals the CNX-013-B2 treated animals maintained their rectal temperature without any further decrease (Figure 4E). At the end of the 75’ exposure body temperature dropped by 4°C in the DIO control animals and only by 2°C in the CNX-013-B2 treated animals. The CNX-013-B2 treated animals appeared to mount a strong adaptive thermogenesis in response to cold exposure. It is important to note that succinate dehydrogenase (SDH) activity increased in gastrocnemius muscle (Figure 4F) and expression of UCP1 was significantly increased in both inguinal and brown fat of CNX-013-B2 treated animals (Figure 4G, H and J). Quantification of UCP1 expression indicated a 45% increase in the number of UCP1 positive cells in the inguinal white adipose tissue of CNX-013-B2 treated animals DIO animals (Figure 4J). We have not yet determined the possible mechanism of CNX-013-B2 mediated increase in UCP1 expression in inguinal fat and brown fat.

### CNX-013-B2 has beneficial effects in liver

**Figure 5:**
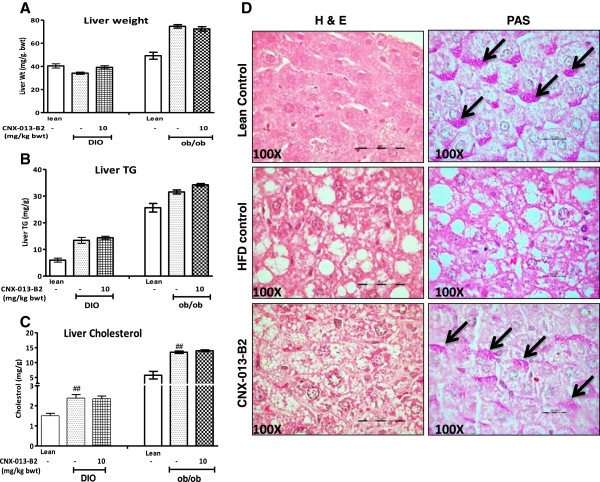
**CNX-013-B2 ameliorates macro vesicular steatosis and increases liver glycogen. A**- Liver weight in DIO and ob/ob mice, **B**- Liver TG in DIO and ob/ob mice, **C**-Liver cholesterol in DIO and ob/ob mice. **D**-Left panel-Hematoxylin-Eosin (H&E) stained sections of liver tissues from DIO mice at 100X magnification, **D**-Right panel-Hematoxylin-Eosin (H&E) stained sections of liver tissues from DIO mice at 100X magnification, periodic acid-Schiff (PAS) stain of liver sections of DIO mice at 100X magnification. Arrows showing glycogen deposits. All the values are expressed as Mean ± SEM; one way analysis of variance followed by Dunnett’s test for representing significance value of the treatment groups. P value significance was represented as (*) <0.05, (**) <0.01 and (***) <0.001 when compared with DIO control.

Treatment with CNX-013-B2 did not increase liver weight in either the ob/ob or in the DIO mice (Figure 5A). Further when compared with their respective controls there was no significant increase in either liver triglyceride (Figure 5B) or liver cholesterol (Figure 5C) content in either the ob/ob or the DIO mice. Histological analysis indicated that the periportal macro-vesicular steatosis, characterized by large lipid droplets and displaced nucleus, observed in the DIO control mice was completely ameliorated by treatment with CNX-013-B2 which instead resulted in pericentral micro-vesicular steatosis characterized by smaller lipid droplets and a centrally placed nucleus (Figure 5D, H&E). Liver glycogen content that had significantly reduced in liver sections from DIO control appeared to have been reasonably restored in liver sections from CNX-013-B2 treated animals (Figure 5D, PAS).

### CNX-013-B2 reduces Cdk5 mediated Phosphorylation of PPARγ

**Figure 6:**
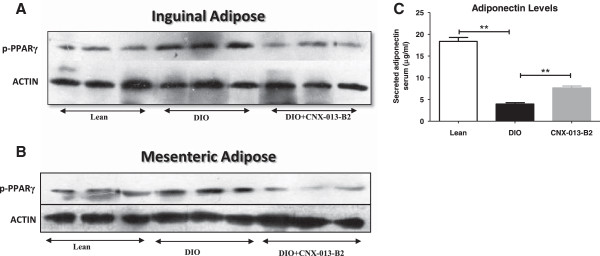
**CNX-013-B2 inhibits Cdk5-mediated Phosphorylation of PPARγ at Ser-273 and also increases serum adiponectin levels.** Inguinal **(A)** and mesenteric **(B)** adipose tissue from DIO study were analyzed with phospho-Ser 273 PPARγ antibodies, normalized to beta-Actin. **C**- Serum adiponectin levels in DIO mice. All the values are expressed as Mean ± SEM; one way analysis of variance followed by Dunnett’s test for representing significance value of the treatment groups. P value significance was represented as (*) <0.05, (**) <0.01 and (***) <0.001 when compared with DIO control.

It is reported that high fat feeding activates protein kinase cdk5 which in turn phosphorylates PPARγ at Serine 273 in adipose tissues [31]. In the DIO control mice phosphorylation of PPARγ at Serine 273 was markedly increased in adipocytes from both mesenteric and inguinal fat depots while in adipocytes from the same depots it was very significantly reduced in the CNX-013-B2 treated animals (Figure 6A, B). Serum adiponectin level, which was reduced in the DIO control animals, was significantly increased in the CNX-013-B2 treated animals (Figure 6C).

### Treatment with CNX-013-B2 induces changes in gene expression in liver, adipose and muscle of C57BL/6 J and ob/ob mice

**Figure 7:**
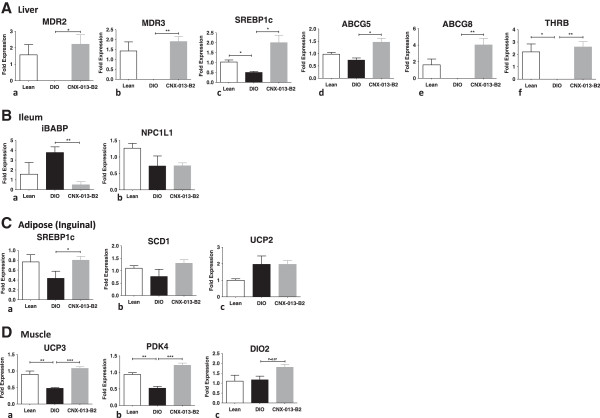
**Gene expression studies after 10 week treatment with CNX-013-B2.** mRNA expression levels of multiple genes from **(A)** Liver, **(B)** Ileum, **(C)** Adipose and **(D)** Muscle were analyzed at the end of the study as mentioned in Materials and methods. All the values are expressed as Mean ± SEM. Statistical comparison between control and treatment group was conducted by unpaired Student’s t test. P value significance was represented as (*) <0.05, (**) <0.01 and (***) <0.001.

**Figure 8:**
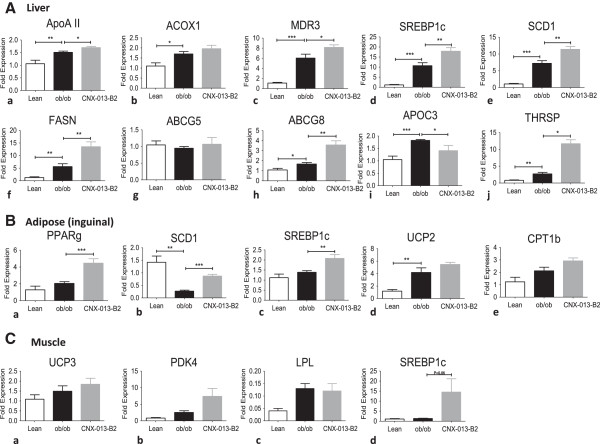
**Gene expression in ob/ob mice after 4 weeks of treatment with CNX-013-B2.** mRNA expression levels of multiple genes from **(A)** Liver, **(** **B** **)** Adipose and **(C)** Muscle were analyzed at the end of the study as mentioned in Materials and methods. All the values are expressed as Mean ± SEM. Statistical comparison between control and treatment group was conducted by unpaired Student’s t test. P value significance was represented as (*) <0.05, (**) <0.01 and (***) <0.001.

To identify the changes in gene expression induced by CNX-013-B2 mRNA expression of a set of genes, that are specific targets of nuclear receptors and are involved in glucose and fat metabolism, was assessed in liver, muscle and adipose from treated animals (Table 2). Treatment with CNX-013-B2 enhanced expression of genes that are reported to be transcribed by RXR/PPARα, RXR/PPARδ and RXR/PPARγ and this was in agreement with the result obtained in cotransfection assays (Table 1). However an increase in expression of genes reported to be transcribed by RXR/LXR heterodimer, in contrast to data from cotransfection assays, was observed in the tissues. While this data can serve as an indirect evidence of activation of RXR/LXR *in vivo* the specific gene expression changes could have been induced by different and overlapping pathways. In absence of data from cotransfection assays we are unable to state if the observed modulation of expression of genes transcribed by RXR/FXR and RXR/TR is a direct impact of CNX-013-B2 or is due to different and overlapping pathways. Treatment for either a short duration of 4 or a longer duration of 8 weeks appeared to induce similar changes in gene expression irrespective of the genetic background and nature of diet (Figures 7 and 8).

**Table 2:** **Specific target genes of nuclear receptors involved in glucose and fat metabolism**

Organ	Heterodimer	Gene	Function	Ref
Liver	RXR/PPARα	ABCB4	Biliary phosphotidyl choline secretion	[32, 33]
		ApoAII	Constituent of HDL-C	[34]
		ACOX1	Peroxisomal fatty acid oxidation	[35]
	RXR/LXR	SREBP1c	Regulation of enzymes of fatty acid synthesis	[36]
		SCD1	Palmitic to palmitoleic and stearic to oleic acid	[37]
		FASN	De novo synthesis of fatty acids	[38]
		THRSP	Lipogenesis	[39]
		ABCG5/ABCG8	Biliary secretion of cholesterol and phytosterols	[40]
Adipose	PPARγ	SCD1	Palmitic to palmitoleic and stearic to oleic acid	[41]
	LXR	PPARγ	Adipogenesis	[42]
		SREBP1c	Regulation of enzymes of fatty acid synthesis	[42]
		SCD1	Palmitic to palmitoleic and stearic to oleic acid	[43]
Muscle	PPARδ	PDK4	Fatty acid oxidation	[44]
		UCP3	Fatty acid oxidation and oxidative stress	[45]

In ileum CNX-013-B2 significantly reduced expression of iBABP (intestinal Bile Acid Binding Protein) involved in intestinal bile acid binding [46] and NPC1L1, critical for intestinal cholesterol absorption [47].In addition CNX-013-B2 also increased mRNA levels of thyroid hormone receptor β (THRB) (Figure 7A:f), and decreased expression of apoCIII (apolipoprotein CIII) in liver, increased expression of DIO2 (De-iodinase 2) and SREBP1c (Figures 7D: c and 8C: d) in muscle.

## Discussion

RXR is often considered ‘sui generis’ owing to its ability to heterodimerize and modulate several other members of the nuclear receptors family. In this report we describe the biological characterisation of CNX-013-B2, a heterodimer selective rexinoid that has been designed, synthesized and developed by Connexios Life Sciences to treat various risk factors of the metabolic syndrome. CNX-013-B2 has ideal pharmacokinetic properties that ensure good distribution specifically into muscle, adipose and liver with a serum half-life of 4-6 hrs in rodents (data not shown). Importantly treatment of mice for 10 weeks did not cause hypertriglyceridemia (Figure 3C and D) or ectopic fat accumulation (Figure 5B; Additional file 2D) or hepatomegaly (Figure 5A) or increase in body weight (Figure 4A, 4D).

In mouse models relevant to obesity and type 2 diabetes, such as ob/ob and C57BL/6 J DIO mice on high fat diet (HFD), treatment with CNX-013-B2 resulted in significant improvement in insulin sensitivity, control of fed and fasting glucose, fed and fasting triglyceride and cholesterol, a reduction in adiposity and body weight gain. The observed whole body insulin sensitivity (Figures 1F, 2E) can be attributed to the peripheral insulin sensitization by CNX-013-B2 and similar improvements in insulin sensitivity have been reported earlier for RXR agonists [48, 49]. There was a significant reduction of p-JNK levels (Figures 1I, 2F), a known mediator of free fatty acids [50] and inflammation [51] induced insulin resistance and a marker of cellular metabolic stress [52]. This coupled with increased p-AKT levels (Figures 1G, 1H, 2G) suggested that treatment with CNX-013-B2 reduced cellular stress and consequently enhanced insulin sensitivity in liver, adipose and muscle. Treatment with CNX-013-B2 ameliorated macrovescicular steatosis in liver (Figure 5D H&E), reduced muscle triglyceride content (Additional file 2D) and also reduced adipose hypertrophy (Figure 4B and C), thus leading to improvement in insulin sensitivity [53]. Such a strong reduction in insulin resistance perhaps, explains the steep reduction in the early phase insulin secretion peak at 10’ time point and 0 – 30’ insulin AUC during OGTT performed in the DIO mice (Figure 1B) and the observed improvement in glucose tolerance in ob/ob mouse model after just 4 weeks of treatment (Figure 2A). Since both fasting and fed insulin levels were not measured early on in this study we are unable to state if the onset of improvement in insulin sensitivity preceded the reduction in fasting glucose levels.

In liver, gene expression profile suggests that a significant part of the pharmacological effect could be due to modulation of RXR/PPARα, RXR/LXR, RXR/THR and RXR/FXR heterodimer complexes. A previous study under similar conditions [54] reported that combined treatment with a PPARα agonist, fenofibrate, and an LXR agonist, T0901317, alleviated insulin resistance and improved glucose tolerance but dramatically exacerbated hepatic steatosis. It is important to note that in CNX-013-B2 treated animals there was no increase in liver triglyceride content in spite of a significant increase in expression of RXR/PPARα and RXR/LXR target lipogenic genes, SREBP1c, SCD1 and FASN [36, 55, 56]. In absence of biochemical data such as oxygen consumption by the animals in the study our attempt to explain the lack of increase in liver triglyceride levels in both DIO on HFD and ob/ob upon treatment with CNX-013-B2 relies mainly on gene expression analysis, weights of adipose depots especially in the DIO mice and serum FFA, TG and glycerol levels. There was an appreciable decrease in weight of adipose depots especially in the DIO mice on HFD (Figure 4B) and yet there was no increase in serum free fatty acid or serum glycerol levels (Additional file 2). Contrary to gene expression profile suggesting increased fatty acid synthesis in liver of CNX-013-B2 treated mice, the DIO mice display amelioration of macrovesicular steatosis (Figure 5D) and in ob/ob mice there is no increase in liver triglyceride levels (Figure 5B). However gene expression profile especially in the muscle and adipose (Figures 7 and 8) suggests enhanced fatty acid oxidation. In absence of additional data we can perhaps state that an increase in fatty acid oxidation in the periphery, including adipose and muscle, is preventing triglyceride accumulation in the livers of CNX-013-B2 treated animals.

Further the role of RXR-PPARα and RXR-LXR heterodimers in mediating the effect of CNX-013-B2 is substantiated by the observed increase in liver glycogen content (Figure 5D) as it has been previously shown in rodent models, that both PPARα and LXR can regulate glycogen synthesis and flux in liver [57, 58].

In adipocytes the insulin sensitizing effects of CNX-013-B2 seems to be mediated by both RXR/PPARγ and RXR/LXR heterodimer complex which is a known insulin sensitizer in adipocytes [59, 60].

In obesity cytokine and high fat diet induced CDK5 mediated phosphorylation of PPARγ is reported to dysregulate expression of a number of genes including adiponectin [31]. A non-agonist PPARγ ligand, SR1664, not only blocked CDK5 mediated phosphorylation but also displayed anti-diabetic activity without causing fluid retention and weight gain [61]. Treatment with CNX-013-B2 also inhibited CDK5 mediated phosphorylation of PPARγ in both mesenteric and inguinal depots and reduced weight of various adipose depots (Figure 6A & B). The enhanced p-AKT levels in mesenteric and inguinal adipocytes and reduced lipolysis suggest that CNX-013-B2 reduced high fat diet induced inflammation leading to better glucose and lipid tolerance.

Among the factors that are implicated in the development of brown adipose tissue, RXRα/γ [62], PPARα [63] and PPARγ [64] are also reported to regulate mRNA expression of UCP1 gene. It therefore appears possible that activation of RXRα/γ, PPARα and PPARγ by CNX-013-B2 could be one of the reasons for the enhanced UCP1 protein expression in the BAT of treated animals (Figure 4H). There are a host of factors, that include transcriptional regulators as well as proteins and secreted mediators, that are reported to regulate browning of white adipose tissue and it is beyond the scope of this article to enumerate role of each of them. However one of the key regulators of browning of white adipose tissue that can be activated by CNX-013-B2 happens to be PPARγ [64]. The browning of inguinal WAT in the DIO mice on HFD appears to be a net result of the regulation of the various pro- and anti-browning factors by the modulation of RXR and its heterodimer partners by CNX-013-B2. Additional experiments are necessary to delineate the mechanism of browning of inguinal WAT by CNX-013-B2.

The activation of RXR/PPARδ in muscle by CNX-013-B2 and increased mRNA expression of UCP3 (Figure 8C:a) could be one of the reasons for the protection from high fat-induced insulin resistance and obesity [45, 65]. It is well known that UCP3 enhances mitochondrial fatty acid oxidation [66] while also decreasing mitochondrial ROS generation [67] and this can perhaps explain the improvement in muscle p-AKT levels (Figure 1G) and succinate dehydrogenase activity (Figure 4F). Succinate dehydrogenase (SDH) activity is considered as an indicator of muscle oxidative capacity [68] and expression of genes involved in oxidative metabolism, including succinate dehydrogenase B, is reported to be reduced in skeletal muscle of diabetes mellitus patients [69]. The increase in SDH activity in treated animals indicates that the oxidative capacity of muscle is significantly modulated by treatment with CNX-013-B2.

In a separate study under progress we have observed an increase in exercise capacity, in terms of treadmill running, of C57BL/6 J mice on HFD after treatment with CNX-013-B2 for 5 weeks (unpublished observations). It will be interesting to examine if activation of RXR/LXR and RXR/PPARδ by CNX-013-B2 in muscle can impact reprogramming of muscle fibers similar to PPARδ overexpression in muscle [45]. Also increase in expression of DIO-2 and UCP3 in muscle [70] and THRB and THRSP [71] in liver suggest that pharmacological effect of CNX-013-B2, observed in this study, could also be due to activation of thyroid hormone signaling in muscle and liver. The expression of genes such as MDR3, ABCG5/8 (in liver) and iBABP and NPC1L1 in intestine suggests modulation of enterohepatic circulation of bile acids leading to inhibition of dietary cholesterol absorption and such an effect has previously been reported for the RXR agonist Bexarotene [72]. Even though the reduction in serum fed cholesterol levels is statistically significant further studies will be necessary to establish that CNX-013-B2 treatment can cause marked inhibition of dietary cholesterol absorption.

The forgoing results provide a pharmacological proof of concept for a selective small molecule-based rexinoid, CNX-013-B2, that combines the lipid lowering effects of PPARα, PPARδ and LXR, insulin sensitizing and glucose lowering effects of PPARα/γ, LXR and THRB and energy uncoupling effects of PPARδ with potential to reduce weight gain. We demonstrate that a coordinated modulation of several nuclear receptors and multiple molecular pathways controlling intermediary metabolism in liver, adipose and muscle has the potential to provide excellent control of metabolic parameters which can address multiple risk factors of the metabolic syndrome.

## Conclusions

In summary, CNX-013-B2 is a selective and orally bioavailable rexinoid. Pharmacological activation of RXR with CNX-013-B2 can give strong glycemic and lipid control, improve insulin sensitivity without increasing body weight, enhance muscle oxidative capacity and function and has a potential to control hepatic steatosis. CNX-013-B2 does not cause hypertriglyceridemia, hepatomegaly and body weight gain. Further preclinical safety and toxicity studies are in progress.

## Electronic supplementary material

Additional file 1: **CNX-013-B2 is a selective rexinoid.** CNX-013-B2 does not activate hRXRα/hLXRα and hRXRα/hRARα heterodimers (A & B). Also CNX-013-B2 does not activate PPAR isoforms alone (C - D). The details of the methods are mentioned in the Materials and methods. (DOC 682 KB)

Additional file 2: **CNX-013-B2 has minimal impact on serum and tissue lipid levels.** After study termination, serum glycerol, free fatty acids and muscle TG were analyzed as mentioned in the Materials and methods. All the values are expressed as Mean ± SEM; one way analysis of variance followed by Dunnett’s test for representing significance value of the treatment groups. P value significance was represented as (*) <0.05, (**) <0.01 and (***) <0.001. (DOC 759 KB)

## Abbreviations

OGTT: Oral glucose tolerance test
HFD: High fat diet
T2DM: Type 2 Diabetes Mellitus
DIO: Diet induced obesity
UCP1: Uncoupling protein 1
SREBP1c: Sterol Regulatory Element-Binding Protein 1c
TG: Triglyceride
LDL: Low-density lipoprotein
FFA: Free fatty acid
NASH: Nonalcoholic steatohepatitis
PBS: Phosphate buffered saline
HOMA-IR: Homeostasis model assessment-estimated insulin resistance.
