# Supplementary material for: CNX-013-B2, a unique pan tissue acting rexinoid, modulates several nuclear receptors and controls multiple risk factors of the metabolic syndrome without risk of hypertriglyceridemia, hepatomegaly and body weight gain in animal models
Source: Diabetol Metab Syndr. 2014 Aug 12;6:83. doi: 10.1186/1758-5996-6-83 (PMC4138375; doi:10.1186/1758-5996-6-83)
Supplement: Supplementary file 1 — Additional file 1: CNX-013-B2 is a selective rexinoid. CNX-013-B2 does not activate hRXRα/hLXRα and hRXRα/hRARα heterodimers (A & B). Also CNX-013-B2 does not activate PPAR isoforms alone (C - D). The details of the methods are mentioned in the Materials and methods. (DOC 682 KB) [file 13098_2014_348_MOESM1_ESM.doc]

**Additional File 1: CNX-013-B2 is a selective rexinoid.**

**A B**

**C D E**

**Additional File 1: CNX-013-B2 is a selective rexinoid.** CNX-013-B2 does not activate hRXRα/hLXRα and hRXRα/hRARα heterodimers (**A &** **B**). Also CNX-013-B2 does not activate PPAR isoforms alone (**C -** **D**). The details of the methods are mentioned in the materials and methods.
