# Supplementary material for: CNX-013-B2, a unique pan tissue acting rexinoid, modulates several nuclear receptors and controls multiple risk factors of the metabolic syndrome without risk of hypertriglyceridemia, hepatomegaly and body weight gain in animal models
Source: Diabetol Metab Syndr. 2014 Aug 12;6:83. doi: 10.1186/1758-5996-6-83 (PMC4138375; doi:10.1186/1758-5996-6-83)
Supplement: Supplementary file 2 — Additional file 2: CNX-013-B2 has minimal impact on serum and tissue lipid levels. After study termination, serum glycerol, free fatty acids and muscle TG were analyzed as mentioned in the Materials and methods. All the values are expressed as Mean ± SEM; one way analysis of variance followed by Dunnett’s test for representing significance value of the treatment groups. P value significance was represented as (*) <0.05, (**) <0.01 and (***) <0.001. (DOC 759 KB) [file 13098_2014_348_MOESM2_ESM.doc]

**Additional File 2: CNX-013-B2 has minimal impact on serum and tissue lipid levels.**

**Additional File 2. CNX-013-B2 has minimal impact on serum and tissue lipid levels.** After study termination, serum glycerol, free fatty acids and muscle TG were analyzed as mentioned in the materials and methods. All the values are expressed as Mean ± SEM; one way analysis of variance followed by Dunnett’s test for representing significance value of the treatment groups. P value significance was represented as (*) <0.05, (**) <0.01 and (***) <0.001.
